# AI-powered fraud and the erosion of online survey integrity: an analysis of 31 fraud detection strategies

**DOI:** 10.3389/frma.2024.1432774

**Published:** 2024-12-02

**Authors:** Natalia Pinzón, Vikram Koundinya, Ryan E. Galt, William O'R. Dowling, Marcela Baukloh, Namah C. Taku-Forchu, Tracy Schohr, Leslie M. Roche, Samuel Ikendi, Mark Cooper, Lauren E. Parker, Tapan B. Pathak

**Affiliations:** ^1^Geography Graduate Group, University of California, Davis, Davis, CA, United States; ^2^Rhizobia LLC, San Francisco, CA, United States; ^3^University of California Cooperative Extension, Davis, CA, United States; ^4^Agricultural Sustainability Institute, College of Agricultural and Environmental Sciences, University of California, Davis, Davis, CA, United States; ^5^Natural Resources Institute, University of Wisconsin-Madison, Madison, WI, United States; ^6^Division of Agriculture and Natural Resources, University of California, Merced, Merced, CA, United States; ^7^California Climate Hub, United States Department of Agriculture (USDA), Davis, CA, United States

**Keywords:** surveys, online data collection, fraud detection, survey farms, AI bots

## Abstract

The proliferation of AI-powered bots and sophisticated fraudsters poses a significant threat to the integrity of scientific studies reliant on online surveys across diverse disciplines, including health, social, environmental and political sciences. We found a substantial decline in usable responses from online surveys from 75 to 10% in recent years due to survey fraud. Monetary incentives attract sophisticated fraudsters capable of mimicking genuine open-ended responses and verifying information submitted months prior, showcasing the advanced capabilities of online survey fraud today. This study evaluates the efficacy of 31 fraud indicators and six ensembles using two agriculture surveys in California. To evaluate the performance of each indicator, we use predictive power and recall. Predictive power is a novel variation of precision introduced in this study, and both are simple metrics that allow for non-academic survey practitioners to replicate our methods. The best indicators included a novel email address score, MinFraud Risk Score, consecutive submissions, opting-out of incentives, improbable location.

## Introduction

In recent years, online surveys have been popular for collecting data efficiently and quickly. Online surveys are thought to offer a convenient and resource-conserving alternative in comparison to paper-based surveys (Dillman et al., 2014). However, this convenience has a downside: an exponential rise in fraudulent responses when the surveys are administered via open distribution links. This rise in fraud responses negatively affects the validity of survey data, undermining the reliability and usability of survey findings. Policy and program decisions are heavily dependent on reliable and valid survey data, and any inaccuracy can have far-reaching consequences in resource allocation, program planning, implementation, and evaluation.

The emergence of Artificial Intelligence (AI) bots and coordinated human fraudsters has made it challenging to maintain accuracy and integrity in online surveys. Bots, or automated software applications simulating human actions, can be programmed to automatically respond to surveys and generate invalid data for financial gain (Kennedy et al., 2020; Storozuk et al., 2020). Bots, capable of submitting thousands of responses within hours, manifest in various forms such as script bots, intermediate bots, and sophisticated bots (Shaw and Cascalheira, 2023). The advent of sophisticated fourth-generation bots poses an even greater challenge, as they rotate through thousands of different Internet Protocol (IP) addresses using real-time adaptation with machine learning to learn the survey's content and accurately mimic human behavior (Shaw and Cascalheira, 2023; Guerar et al., 2021).

Survey farms, comprising coordinated malicious human actors, collaborate to exploit survey incentives, introducing carefully constructed responses that distort data (Ofir, 2019; Pozzar et al., 2020). Human fraudsters can work together with bots for all or a portion of their survey submissions as bots can be trained with human input (Brainard et al., 2022). Studies have revealed highly active IPs that exhibit a systematically organized approach to survey completion, depicting survey taking as a cottage industry (RepData, 2021). Consequently, survey professionals relying on open distributions for data collection must implement robust strategies to detect and mitigate fraudulent responses.

Fraudulent responses can distort accurate representation of the valid sample (Kennedy et al., 2020; Godinho et al., 2020; Pratt-Chapman et al., 2021), misleading survey professionals, researchers, and decision makers alike (Kennedy et al., 2021). Online survey fraud can have even larger effects on data collected from historically underserved and marginalized groups; while online surveys can reach such populations that may be otherwise difficult to reach (Bybee et al., 2022; Goodrich et al., 2023; Griffin et al., 2022), fraudulent responses exacerbate the challenge of obtaining authentic data from groups already facing significant participation barriers (Dewitt et al., 2018).

In light of these concerns, this study contributes to the existing knowledge base by offering practical strategies to identify fraudulent responses within online surveys. By equipping survey professionals with the tools to detect fraudulent data, we can reinforce the validity of survey findings. Our investigation focused on evaluating 31 distinct indicators and six ensembles drawn from 1,944 responses from two separate surveys that targeted farmers and ranchers (henceforth referred to as “producers”) in California during 2023. Analyzing these datasets, we dive into the implications of fraud on survey integrity, data accuracy, and time allocation. In addition, we highlight the most effective fraud indicators identified across the two surveys, including a novel email address scoring technique. The implications of this study extend beyond the realm of survey methodology. They underscore the importance of research integrity and data validity in an era where online surveys are increasingly used (Zickar and Keith, 2022) to understand complex social phenomena and inform evidence-based decision making.

### Strategies for addressing survey fraud: a literature review

We reviewed 48 peer-reviewed journal articles published between 2015–2023 that documented online survey fraud and the countermeasures employed. Our search for relevant publications involved exploring academic citation indexes and examining references cited in other research articles.

While numerous strategies emerge in the battle against online survey fraud, the key findings of our literature review underscore the insufficiency of any single strategy alone. Instead, a comprehensive approach involving multiple strategies before, during, and after survey distribution is imperative (Shaw and Cascalheira, 2023; Dewitt et al., 2018; Lawlor et al., 2021; LePine et al., 2023).

**Figure 1:**
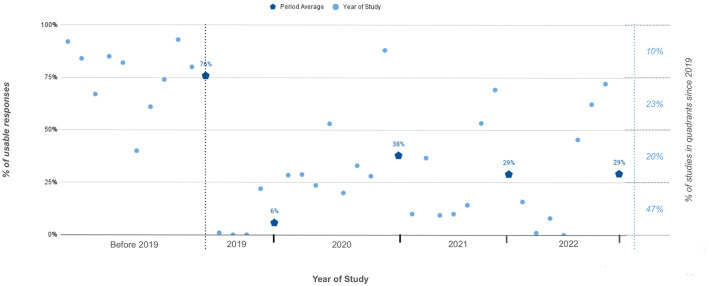
Percentage of usable responses to online surveys from 36 studies published before and after 2019.

Our review, as shown in Figure 1, reveals a decline in the percentage of usable responses in online surveys overtime. Tactics once effective, such as CAPTCHA (Teitcher et al., 2015), hidden or “honeypot” questions, geolocation (Kennedy et al., 2020; Levi et al., 2022), speeding detection (Guest et al., 2021), and attention checks (Oppenheimer et al., 2009), are now becoming less reliable or even obsolete (Kennedy et al., 2021; Ballard et al., 2019; Campbell et al., 2022; Kantar, 2023; Zhang et al., 2022). Fraudsters adapt quickly, with the reliability of chosen strategies even changing within a single study (Shaw and Cascalheira, 2023; Dewitt et al., 2018; LePine et al., 2023; Campbell et al., 2022; Moss et al., 2021). This evolution reflects fraudsters' increasing sophistication with the structure, content, and domain of individual surveys over time (Dewitt et al., 2018).

Of 48 publications reviewed, 36 explicitly stated the percentage of fraud responses received in their total study sample. The year of study was based on the year when the survey was conducted, if the information was missing we used the year prior to the publication year. Fifteen publications used in this figure are not listed in the *References*.

While the documentation of fraud in online surveys dates back to 2005, primarily identified as “multiple submissions” (Dewitt et al., 2018; Lawlor et al., 2021), a notable escalation in fraud prevalence has been observed since 2018–2019 (Moss et al., 2021). This surge can be attributed to the growth of professional survey takers associated with survey panels on Amazon Mechanical Turk (Mturk) (Kennedy et al., 2020). The online platform enables crowdsourcing of small tasks and has led to a troubling decline in usable survey responses (Figure 1). In addition to these factors, research indicates that a significant proportion of fraudulent survey responses originate from regions with notable economic disparities, such as India and Venezuela. This highlights how global inequalities can also drive the prevalence of survey fraud (Kennedy et al., 2021; Moss et al., 2021). In the pre-2019 era, online survey responses typically expected a usability rate of more than 75%. However, in today's landscape, only 10% of studies using open online surveys can anticipate such high usability. Post-2019, response usability has predominantly lingered within the 0%−25% quartile (Figure 1). What was once an incidence of fraud ranging from 14 to 18% (Chandler and Paolacci, 2017) has surged to around 80%, with documented instances reaching 100% (Shaw and Cascalheira, 2023; Pozzar et al., 2020; Lawrence et al., 2023). This indicates not only an increase in fraud over time, but also a decrease in the effectiveness of previously reliable fraud deterrence and detection strategies.

Despite these challenges, the existing literature contains a multifaceted toolkit to enhance data integrity to help survey professionals. The most successful cases against fraud have involved complex, multi-phased (Hardesty et al., 2023) distribution and processing plans (Glazer et al., 2021). The essential process of data cleaning and processing has been reported to take between 20–120 h and between 1–2 months (Shaw and Cascalheira, 2023; Bybee et al., 2022; Dewitt et al., 2018; Lawlor et al., 2021; Lawrence et al., 2023; Glazer et al., 2021). Key strategies used include open-ended questions, manual verification of personal information, domain knowledge or expert validation, logical consistency, and browser fingerprinting.

The most frequently employed strategy within the fraud detection literature is the manual examination of open-ended responses. However, questions must be meticulously constructed, considering the presence of diligent human fraudsters or AI-powered bots with sophisticated programming. Ongoing manual techniques during recruitment, such as verifying participant eligibility and personal information (such as reviewing uploaded identification cards or mailing addresses), have proven highly effective (Godinho et al., 2020; Glazer et al., 2021; Bell et al., 2020). A less time-consuming approach involves checking for the consistency of information (Ballard et al., 2019) presented in different ways (age, gender, location, etc.) as well as logical consistency between answers. The use of domain knowledge or expert validation questions, which only individuals with specific knowledge in the surveyed domain can accurately answer, stands out as least labor intensive to process and increasingly effective even as fraudsters evolve (Pozzar et al., 2020; Goodrich et al., 2023; Zhang et al., 2022; Mitchell et al., 2020).

In the realm of online advertising, addressing click fraud—characterized by repeatedly clicking advertisements to generate revenue—has parallels with online survey fraud (Sadeghpour and Vlajic, 2021). Detection techniques involve distinguishing between the behavioral characteristics of bots, fraudsters and valid respondents. To achieve this, the availability of verified data to compare against potentially fraudulent data becomes crucial (Ilagan and Falk, 2022). Consequently, an important consideration to reduce fraud is to control survey distribution by disseminating surveys through verified email lists and limiting survey dissemination on social media platforms (Wardropper et al., 2021). All and all, having fraud detection algorithms in place before data collection is recommended (Ballard et al., 2019; Wardropper et al., 2021). Survey professionals should remain vigilant, regularly evaluating the effectiveness of their fraud detection strategies (Godinho et al., 2020; Ballard et al., 2019).

We note four significant limitations in the existing literature, which consists of largely anecdotal studies. These studies typically: (1) rely on experiences from a single online survey and offering recommendations, (2) do not rely on a verified sample, and thus are (3) without robust empirical support from which they can draw replicable conclusions (Shaw and Cascalheira, 2023; Kennedy et al., 2021). Additionally, there is a scarcity of research that (4) investigates differences in fraud sophistication levels between overlapping surveys and how detection strategies might be more or less effective depending on the type of fraud involved. Previous studies may underestimate the occurrence and diversity of fraudulent tactics, and the viability of existing detection strategies, highlighting the need for a more systematic and comparative analysis.

In this paper, we build on the existing survey fraud detection literature by (1) comparing two surveys (2) relying on a two-step process to independently verify our samples for testing, and (3) systematically examining which fraud detection strategies are most effective, eliminate the greatest amount of fraud, and can be implemented with reasonable confidence without excluding legitimate respondents. Moreover, when multiple surveys are examined with (4) differing levels of fraud sophistication, variations in the effectiveness of fraud detection strategies are made evident.

Building upon the groundwork laid by Zhang et al. (2022), who measured the precision and recall of 28 common fraud indicators and six indicator combinations systematically, our study expands on their approach by utilizing empirically verified samples of responses collected through verified contact lists, and comparing this to responses collected through more open distribution channels. Furthermore, we introduce new indicators and explore the performance of indicators beyond the context of Mturk.

## Methods

This section provides a comprehensive overview of the methodologies employed for sample acquisition and verification, along with an in-depth exploration of the tested survey fraud indicators and the assessment methods applied to evaluate their effectiveness.

We systematically examined and implemented 31 fraud indicators, 27 drawn from existing literature and 4 not previously documented. Indicators were examined across two distinct surveys distributed through both closed and open distribution channels. Given how increasingly difficult it is to reach our target population via online surveys, we needed an approach that prioritized keeping all possible valid responses without contaminating our sample with fraudulent responses. Because we examined two surveys, we could compare the quality of fraud tests within different contexts.

In addition, we introduce two approaches new to the fraud detection literature, an *email address score* and a *post-submission response verification protocol*. The first approach assigns points based on the structure of an email address, offering a swift and efficient method to identify potentially fraudulent email addresses. The second approach entails sending emails to respondents and requesting verification of specific survey responses submitted.

Thirdly, we aimed to use performance metrics that could be easily understood and implemented by non-academic survey practitioners who might not have advanced statistical skills. We opted for precision and recall, introducing a novel variation of precision we call predictive power that allows for a direct comparison of indicators across imbalanced samples.

Finally, by conducting two consecutive surveys with the same population in the same year, we were able to improve iteratively and test techniques based on what we learned from (1) the first survey; (2) what we continuously uncovered through reviews of documents, websites, and literature; and (3) conversations with survey professionals including market research firms.

### The two surveys

#### Survey 1—climate needs assessment

A 30-min survey with 28 questions to identify the climate impacts, response strategies, and information needs of California producers to climate change (Ikendi et al., 2024). The survey utilized a direct email invitation to a verified list of 14,933 producers and an open link distributed through industry contacts and social media. The list used was DTN's FarmMarketID, a leading farmer contact curator recommended by a recent review of public and private sample sources in agriculture (Ulrich-Schad et al., 2022). There was a $50 lottery incentive for the first 10 respondents from both distributions. After removing incomplete, non-consenting, and disqualified respondents, the survey received 1,922 responses over 91 days between February and May 2023. For the purpose of fraud research, 560 of 1,540 responses from open distributions were verified as fraudulent and all 382 responses from closed distributions were verified as valid. The remaining 1,080 were left as potentially fraudulent. The study was approved by the UC Davis Institutional Review Board (IRB) ID 1841798-2.

#### Survey 2—wildfire impacts and response

A 30-min survey with 34 questions to identify California producers' impacts from and response strategies to wildfires (Pinzón et al., 2024a,b). The survey utilized a direct invitation to a verified list of 19,518 producers and an open link distributed as three separate traceable survey copies in three venues: a social media programmatic campaign, industry newsletters, and word of mouth. The list included DTN's FarmMarketID, and California's Certified Organic Farmers list. There was a $20 incentive for all qualifying respondents and inclusion into a $200 lottery for eight winners. After removing incomplete, non-consenting, and disqualified respondents, the survey received 2,088 responses over 113 days between April and August 2023. For the purpose of fraud research, 627 of 1,616 responses from open distributions were verified as fraudulent and all 471 responses from closed distributions were verified as valid. The remaining 989 were left as potentially fraudulent. The study was approved by the UC Davis Institutional Review Board (IRB) ID 1764124-1.

### Sample verification for fraud tests

To create an independently verified sample for testing, we needed to confidently differentiate between fraud and valid responses. To do so, we employed a two-step process. First, we distributed one-use personal survey invitation links to verified lists via direct email through Qualtrics (i.e. closed distributions). This method ensured a valid sample sourced from verified members of our population before the survey's broader distribution for both Survey 1 and Survey 2. To establish our fraud sample, we disseminated reusable, open links to both surveys through social media and industry newsletters (i.e. open distributions), then conducted a manual review of these responses' open-ended questions to identify fraudulent submissions. Responses that could not be confidently discerned as fraudulent based on their open-ended responses were contacted via email. Those that remained unverified were categorized as *potentially fraudulent* and excluded from our fraud test analysis.

#### Manual review of open-ended questions

Our manual review process involved an examination of responses to open-ended questions, specifically tailored to uncover patterns, vocabulary, and discourse characteristics pertinent to California farming and ranching demographics. This analysis, encompassing ~70 h for both surveys, considered responses exhibiting evident suspicious traits, such as duplicates and nonsensical or illogical content. Suspicious responses that did not conclusively indicate fraud were maintained as *potentially fraudulent*. We examined the most frequently used terms across each survey and differentiated linguistic cues distinguishing fraudulent from legitimate responses, including uncommon grammatical errors and terminology (e.g. “plantations” and “typhoons”). Distinct response patterns emerged between fraudulent responses between Survey 1 and Survey 2.

#### Fraudulent responses in Survey 1

The majority of fraudulent responses in Survey 1 displayed textbook-like verbosity, plagiarism, formality, repetition, and even duplication from the survey's content, indicative of automation from AI-bot-generated content. Notably, a specific question, “*What are your top three climate concerns?”* with three short answer boxes, proved highly effective in capturing fraudulent responses. Even seemingly credible answers, like “*increased salinization*,” “*increased drought levels,”* and “*high temperatures leading to increased crop losses*,” were identified as fraudulent when repeated in a different order by multiple respondents. Closer examination revealed that many seemingly credible responses were copied from segments of the survey itself, suggesting the presence of bots learning from the survey. In contrast, valid responses in Survey 1 exhibited emotional resonance, brevity, and an informal tone, contrasting with the more formulaic and academic responses within the fraud responses. We verified 30% (*n* = 460) of submissions to the open distribution as fraudulent using open-ended responses alone.

#### Fraudulent responses in Survey 2

In Survey 2, the distinction between fraud and legitimate responses became much less apparent, as fraudulent responses demonstrated greater relevance, subtlety, and consistency compared to those in Survey 1. For instance, one respondent mentioned, “*The fire started from the dry grass on my field and moved through to the wooden structure in my farm,”* but was later identified as fraudulent due to providing an identical answer as another respondent (duplication). Another respondent stated, “*I've had enough of this. It's a vicious circle!”* when referring to the wildfire impacts on their livestock (caught by uncommon word choice and duplication). Fraudulent respondents in Survey 2 connected their answers between questions, effectively emulating genuine respondents (see Supplementary material S1 for a detailed example).

Survey 2's fraudulent responses exhibited a level of authenticity, emotion and consistency unseen in Survey 1, making differentiation exceedingly difficult. Two hundred respondents with open-ended responses remained indiscernible as conclusively fraudulent. Consequently, we could only verify 10% (*n* = 159) of submissions to open the distribution as fraudulent using open-ended responses alone. Compounding the issue, 67% (*n* = 763) of respondents left all open-ended questions blank. To overcome this challenge, we emailed participants for verification.

#### Post-submission response verification protocol (Survey 2)

In an effort to improve the verification of responses in Survey 2 we requested specific details related to three questions they answered in their original survey submission 3–6 months before (production type, zip code, wildfire years). We received 104 bounces and 560 responses, of which all but one were fraudulent (our method to determine this is described below). This approach enabled us to validate an additional 473 fraudulent responses for analysis.

Remarkably, among the fraudulent responses received, 82% (*n* = 442) accurately confirmed at least one of the three survey questions, and 28% verified all three. Despite this, patterns in their greeting, sign-off, response structure and duplication between respondents indicated their fraudulent nature. While previous studies have attempted to contact potentially fraudulent respondents via email, the majority have documented a complete lack of responses (Shaw and Cascalheira, 2023; Pratt-Chapman et al., 2021; Bybee et al., 2022; Ballard et al., 2019; Lawrence et al., 2023; Velo Higueras, 2023). None of these previous studies have asked fraudsters to verify specific survey data as we did. This observation suggests an evolution in the sophistication of survey fraud groups and their tools, indicating that some groups are keeping records of their responses for multiple months.

### Fraud and validity indicators tested

After verifying fraud and valid responses from both surveys, we tested 31 indicators and six ensembles across two samples—one from each survey, each consisting of manually categorized fraud and valid subsamples. We examined the frequency of each indicator and ensemble appearing within each subsample.

**Tests 1–3, Speeding:** completing the survey within a period less than percentage of the median time taken by authentic respondents (Jibunu, 2021). Speeders were flagged as either Tier 1 (≤ 30% of the median time; Test 1), Tier 2 (31%−50% of the median time; Test 2), or Tier 1 and 2 (≤ 50% of the median time; Test 3).

**Tests 4–5, Timestamps:** Test 4 identified responses with identical durations in seconds. Test 5 flagged survey responses that commenced at or after 12:00 am and before 5:00 am local time, recorded in Pacific Standard Time (PST).

**Tests 6–9, Consecutive:** Tests 6–9 were conducted on the time between two successive survey submissions to identify those that either began or ended in a series. Submissions were flagged if they started within 1 min of another submission's starting (Test 6), ended within 1 min of another submission ending (Test 7), started within 1 min before *and* after another submission's starting (Test 8), or ended within 1 min before *and* after another submission ending (Test 9). While consecutive end times are a recognized indicator in the literature (referred to as consecutive submissions), the use of consecutive start times represents a novel indicator that, to our knowledge, is not documented in the existing literature.

**Tests 10–11, Geolocation:** examined the respondent's location based on their IP address. Respondents with IPs outside of the US (Test 10) or outside of the study region of California (Test 11), were flagged.

**Test 12–14, Suspicious activity:** involved testing IPs for their suspicious nature using three available tools: (1) IPHub was used to identify virtual private network (VPN) use (Test 12), (2) MaxMind was employed to assess the MinFraud Risk Score associated with the IP and email address is provided (Test 13), and (3) MxToolbox was used to check if the IP was connected to a blacklisted server (Test 14). Maxmind's MinFraud Risk is an industry-standard and paid service ($0.05 per query) that can analyze fraud risk based on IP and contact information. All flagged responses had a score above 59, indicating a 59% likelihood of being fraudulent. All three services necessitated programming by the research team, making these tests reliant on certain technical skills.

**Tests 15–17, Duplication:** Identified duplication across the IP address collected by Qualtrics (Test 15) and across two survey questions, email address (Test 16), and phone number (Test 17).

**Tests 18–20, Data quality:** Test 18 checked for patterned responses to matrix questions, including straight-lining and zigzag patterns. Tests 19 and 20 involved two attention checks: the first, “*Still with us? Please check ‘Neutral or Unsure' for this statement”*, was early in the survey (question 11) and placed within a likert-scale question (Test 19). The second, “*…Still with us? Please check this box!”*, was toward the end (question 21) and within a lengthy multiple-choice question (Test 20).

**Test 21, Logical inconsistencies:** These are one or more answers from the same respondent that are contradictory, mutually exclusive, or illogical. Test 21 flagged mutually exclusive sets of responses: (1) selecting responses such as “I am a first generation” *and* “I am multi-generational” farmer, or (2) reporting that they have been farming more years than they have been alive.

**Tests 22–25, Improbable:** We identified responses deviating from the anticipated characteristics of our study population as potential fraud indicators. The tests encompassed various criteria:

- Test 22: Location (non-CA): detecting zip codes outside our study region of California.
- Test 23: Location (urban): flagging zip codes within the inner city of major metropolitan areas, like Los Angeles, where our farming population is less likely to be.
- Test 24: Demographics: Identifying uncommon demographics, such as selecting “white” race along with descriptors like “socially disadvantaged.” Additionally, looking for specific racial categories chosen at a much larger proportion than the populations being studied.
- Test 25: Reverse scored: A section of Survey 1 asked respondents about their climate adaptation practices and information requirements. If respondents expressed being “not at all interested” in implementing a practice but indicated a “high need” for information about that specific practice more than twice, then they were flagged.

**Tests 26–27, Consistency and domain knowledge checks:** Consistency checks verify the consistency of responses provided by a participant across different parts of the survey. Domain knowledge checks involve survey questions that determine if a respondent possesses expertise in the specific domain of the survey. In Test 26, respondents selected their counties from a multiple-choice list of 58 counties and then provided their zip code on the next page. Responses were flagged if the selected county did not match the entered zip code. For Test 27, participants were initially asked about the crops or livestock they grow and later presented with a similar question but with a different answer choice conveying the same meaning (e.g., “sheep” producers should specify they have “ruminants” or “grazing animals”). This test not only assessed respondents' knowledge of their crops or livestock but also their consistency in matching responses to similar questions.

**Test 28, Email address score:** This was identified by looking for repetitive and discernable patterns in the structure of email addresses, such as proper name capitalization, recurring names or segments, profanity, and permutations of the same structural elements. Observing a discernible pattern in the length and numerical composition of email addresses led us to develop an email score. The score was created by comparing a dataset of valid and fraudulent emails for patterns; responses with a score of 2 or more were flagged (more details explained in *Results and discussion)*. To our knowledge, this is a novel approach to email address analysis, not yet documented in the literature. The formulas for identifying fraud email addresses are provided in the data repository, allowing for easy replication and application of the developed method.

**Test 29, Email bounced:** All respondents that included email addresses were checked using an email address verification tool (emaillistverify.com), emails that bounced were flagged.

**Tests 30–31, Incentives:** Respondents who either opted out of the survey incentives (Test 30) or requested to receive their incentive through Zelle or Venmo (Test 31) were marked as *not* fraudulent. Zelle and Venmo are two payment platforms that are difficult to access outside of the US, as they require a US bank account (Zelle, 2023), or US phone number and location (Venmo, 2022). Additionally, incentives are tedious to process through these platforms, since unlike Paypal and Amazon, they do not yet have integrations for sending incentives. The use of Zelle/Venmo represents a novel indicator that, to our knowledge, has not been documented in existing literature.

**Ensembles 1–6:** Ensembles 1, 2, and 3 combined indicators additively, whereby the ensemble would mark a response as fraud if it had been flagged by any one of the indicators used by that ensemble. Ensembles 4, 5 and 6 combined groups of indicators using simple logical operators (i.e. AND, OR, etc.) in order to achieve a more sophisticated fraud test that harnessed the predictive power of several indicators without accumulating as much error.

### Determination of indicator quality

To evaluate the quality of each indicator, we initially computed its *predictive power*—a slight modification to *precision* that has significant advantages explained below—and *recall*, subsequently utilizing these values to assign a quality score. Precision and recall are commonly used metrics in evaluating the performance of fraud detection systems (Mohan et al., 2024; Alfaiz and Fati, 2022), and they are easy to implement and replicate by non-academic survey practitioners without advanced statistical and programming expertise. We therefore use these metrics to assess each fraud indicator, and explain them below.

Predictive power is a variation of precision that takes into account the proportion of fraud in the sample. Both predictive power and precision are measures of predictive accuracy, or the likelihood that a response is correctly flagged as fraud, but predictive power **uses percentages instead of raw counts for the number of responses flagged** (see below for a comparison of the two formulas). Recall (also referred to as sensitivity or the true positive rate) measures the quantity of fraud flagged and is given as a percentage of total fraud. We used the standard formula for recall:

**Recall** = **True Positives/Total Positives**, where True Positives = # fraud flagged; and Total Positives = total # of fraud responses.

Predictive power is calculated as follows:

**Predictive Power** = **True Positive Rate/(True Positive Rate + False Positive Rate)**, or equivalently,

**Predictive Power** = **(% of fraud flagged)/(% of fraud flagged + % of valid flagged)**, where % of fraud flagged = # of fraud flagged/total fraud responses, and % of alid flagged = # of valid flagged/total valid responses.

For reference, the standard formula for precision (sometimes referred to as positive predictive value or PPV) is as follows:

**Precision** = **(True Positives)/(True Positives + False Positives)** Or equivalently, **Precision = (# of fraud flagged)/(# of fraud flagged + # of valid flagged)**.

We developed predictive power as a novel variation of precision in order to more easily and consistently compare fraud indicators across imbalanced samples by accounting for the proportion of fraud in the sample. To demonstrate this, let us consider two hypothetical survey samples A and B. A contains 80% fraud (20% valid responses), and B contains 30% fraud (70% valid responses). Now imagine we have a useless fraud indicator that flags responses at random without effectively differentiating between fraud and valid. The precision of this indicator, which operates purely by chance, would nonetheless vary in direct proportion to the percentage of fraud in the sample (Table 1). So for sample A, which contains 80% fraud, the precision of our chance indicator would be near 0.8, and for sample B, which contains 30% fraud, the precision would be around 0.3. Since the traditional precision formula uses only the raw counts of true positives and false positives, it will simply yield the percentage of flagged responses that are fraud (true positives), which for our chance indicator—essentially a random subsample generator—should be roughly representative of the sample as a whole. By contrast, predictive power (PP) compares the *rate* at which an indicator correctly flags a fraudulent response as fraud (the true positive rate, or TPR) to the *rate* at which valid responses are flagged as fraud (the false positive rate, or FPR). If the TPR and FPR are equal—meaning the indicator does not distinguish between fraud and valid, as is the case with our hypothetical chance indicator—then PP would be 0.5. If fraud is flagged at a higher rate (TPR > FPR), then PP would be above 0.5 (maximum = 1), and if *valid* responses are flagged at a higher rate (FPR > TPR), then PP would be < 0.5 (minimum = 0). The chance indicator would thus have a PP of close to 0.5 for both samples, a value which rightly corresponds to the 50–50 chance of correctly identifying any one response as fraud (when the proportion of fraud in the sample is ignored). On the other hand, the vastly different values yielded by precision for the imbalanced samples A and B are deceptive (0.8 for A, and 0.3 for B). They could be mistaken to imply better or worse performance of an indicator that is totally blind to its target. If these precision values are used to assess indicator performance, they should be read alongside the proportion of fraud in the sample lest we risk misinterpretation. To conclude, PP is a useful alternative to precision because it allows us to consistently measure the predictive utility of any given indicator across multiple imbalanced samples, as well as to more easily identify those that outperform chance.

**Table 1:** Precision vs. predictive power for a chance indicator.

**Fraud indicator**	**Sample A**		**Sample B**	
	**Total fraud** = **80, total valid** = **20**		**Total fraud** = **30, total valid** = **70**	
	**Precision**	**Predictive power**	**Precision**	**Predictive power**
Chance indicator^*^	0.8	0.5	0.3	0.5

^*^A chance indicator in this example is an indicator that flags responses at random without differentiating between fraud and valid.

Based on the performance of each indicator in correctly identifying fraud as assessed by its predictive power, we assigned each a categorical score ranging from *ineffective* to *very high* (Table 2). Our quality score was based primarily on the predictive value of each indicator, but we considered recall for indicators that were on the cusp (e.g. near the threshold between *poor* and *moderate*). When an indicator was near the cusp, a recall above 25% would result in an upgrade (e.g. from *poor* to *moderate*), whereas a recall below 5% would result in a downgrade.

**Table 2:** Indicator quality thresholds.

**Indicator quality**	**Predictive power**
Very high	≥0.95
High	≥0.90
Moderate	≥0.80
Poor	≥0.70
Ineffective	< 0.70

Finally, we examined which indicators, when used in combination, would yield the best results. We called these indicator ensembles and created them by combining individual indicators with high predictive power, leaving out indicators that were specific to our survey population and thus not easily usable outside of our discipline. In addition to measuring predictive power and recall, we also examined error and residual fraud to more fully account for their effectiveness in cleaning the survey samples of fraud amongst six indicator ensembles. Error refers to the percentage of valid responses incorrectly flagged by the ensemble, and residual fraud measures what percentage of the remaining sample is fraud after running an ensemble to remove suspected fraud.

## Results and discussion

In this section, we report and analyze our research results, exploring a range of fraud indicators that offer insights into detecting and addressing fraudulent responses in online surveys. Table 3 presents the predictive power, recall, error and literature corroboration of each of the 31 indicators tested across two surveys.

**Table 3:** Evaluation of fraud and validity indicators across two surveys with literature corroboration.

**Indicator tests**					**Survey 1**				**Survey 2**			
**ID**	**Category**	**Fraud indicator**	**Overall indicator quality**	**Lit. corroboration**	**Predictive power**	**Recall (fraud)**	**Error (valid)**	**Indicator quality**	**Predictive power**	**Recall (fraud)**	**Error (valid)**	**Indicator quality**
						***n** =* **460**	***n** =* **382**			***n** =* **627**	***n** =* **471**	
1	Speed	Speeder, tier 1	Variable	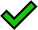	0.89	2%	0.3%	Moderate	1.00	0.5%	0.0%	Very high
2	Speed	Speeder, tier 2	Variable	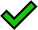	0.70	8%	3%	Ineffective	0.87	31%	5%	Moderate
3	Speed	Speeder, tier 1 or 2	Variable	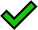	0.73	10%	4%	Poor	0.87	32%	5%	Moderate
4	Timestamps	Identical duration	Poor	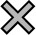	0.70	36%	15%	Poor	0.81	55%	13%	Moderate
5	Timestamps	Start time *(12–5am)*	High	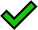	0.96	50%	2%	Very high	0.92	14%	1%	High
6	Consecutive	Start (< 1 min)	Moderate	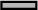	0.80	90%	23%	Moderate	0.79	72%	19%	Poor
7	Consecutive	End (< 1 min)	Moderate	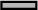	0.83	89%	18%	Moderate	0.84	70%	14%	Moderate
8	Consecutive	Start (< 1 min before/after)	High	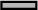	0.93	59%	4%	High	0.98	57%	1%	Very high
9	Consecutive	End (< 1 min before/after)	Very high	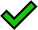	0.94	58%	3%	Very high	0.99	53%	1%	Very high
10	Geolocation	Non-US IP	Moderate	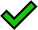 + 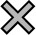	0.87	2%	0.3%	Moderate	0.87	7%	1%	Moderate
11	Geolocation	Non-California IP	Variable	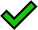	0.91	58%	5%	High	0.71	41%	17%	poor
12	Suspicious activity	VPN (IP Hub)	Poor	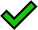 + 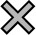	0.72	7%	3%	Poor	0.88	18%	2%	Moderate
13	Suspicious activity	MinFraud Score	Very high	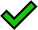	1.00	38%	0%	Very high	1.00	38%	0.0%	Very high
14	Suspicious activity	BlackList (MxToolbox)	Ineffective	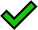 + 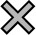	0.51	50%	47%	Ineffective	0.50	52%	53%	Ineffective
15	Duplication	Duplicate IP	Moderate	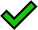	0.86	33%	5%	Moderate	0.93	47%	3%	High
16	Duplication	Duplicate email address	Variable	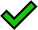	0.93	3%	0.3%	High	0.78	3%	1%	Poor
17	Duplication	Dup phone number	(Very high)	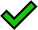	0.96	6%	0.3%	Very high	–	–	–	–
18	Data Quality	Straighlining or zigzag	Ineffective	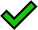 + 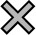	0.70	8%	3%	Poor	0.44	36%	46%	Ineffective
19	Data Quality	Attention check (early)	(Ineffective)	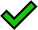	–	–	–	–	0.53	8%	7%	Ineffective
20	Data Quality	Attention check (late)	(Ineffective)	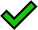	–	–	–	–	0.48	26%	28%	Ineffective
21	Logics	Mutually exclusive	Ineffective	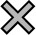	0.70	3%	1%	Ineffective	0.79	4%	1%	Poor
22	Improbable	Location (non-CA zip)	High	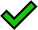	0.99	42%	1%	very high	0.94	10%	1%	High
23	Improbable	Location (urban zip)	Very high	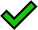	0.61	4%	3%	Ineffective	0.98	12%	0%	Very high
24	Improbable	Demographics	(High)	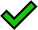	0.90	25%	3%	High	–	–	–	–
25	Improbable	Reverse scored	(Moderate)	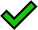	0.83	5%	1%	Moderate	–	–	–	–
26	Consistency	Zip code—county	(Poor)	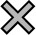	–	–	–	–	0.78	8%	2%	Poor
27	Consistency	Expert validation check	(Ineffective)	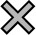	–	–	–	–	0.26	9%	24%	Ineffective
28	Email	Email address score	Very high	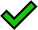	1.00	60%	0.3%	Very high	0.97	22%	1%	Very high
29	Email	Email bounced	(Variable)	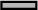	1.00	1%	0%	very high	0.62	4%	2%	Ineffective
30	Incentives^a^	Opted out	Very high	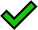 + 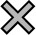	0.98	32%	1%	Very high	0.99	34%	0%	Very high
31	Incentives^a^	Venmo/Zelle request	(Very high)	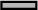	–	–	–	–	1.00	16%	0%	Very high

For fraud indicators, predictive power gauges the success rate of an indicator in identifying fraud, with a value of 1 representing a perfect success rate and 0.5 being equivalent to chance. For instance, in Survey 2, the first indicator, Speeder Tier 1, accurately identified 0.5% of fraud cases without incorrectly flagging any valid cases (valid = 0.0%), resulting in a predictive power of 1. The percent of fraudulent and valid responses flagged by each indicator are represented by recall and error, respectively. The “n=” denotes the total number of cases verified for analysis and categorized as fraud and valid. Cells with a dash “–” signify areas where data was not obtained because the indicator was not available for the survey.

Indicator quality is determined first by predictive power and then fine-tuned by recall (described in Methods). Determinations in the overall indicator quality column is based on a comparison of that indicator's quality between both surveys. Overall indicator quality is marked variable when there was a difference of two or more ranks between the quality assessments in both surveys. Parentheses denote testing limited to one survey.

The literature corroboration column indicates whether our findings corroborated the existing literature “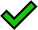,” contradicted the existing literature “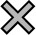,” both “
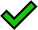
+ 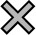,” or conducted novel checks not previously discussed in the literature “
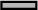
”.

^a^The last two rows examine the two indicators that specifically flagged valid responses. For these validity indicators, predictive power measures the success rate of flagging valid responses, recall measures the retrieval of valid responses, and error the retrieval of fraudulent responses.

We find that no individual indicator employed in isolation demonstrated both a strong predictive power (>0.90) and equally high recall of fraud (>90%). Only indicator ensembles achieved these results, albeit with persistent unacceptable error rates exceeding 5%. Additionally, we find that several indicators exhibit variations in quality between surveys. We suspect this latter finding had to do with the varying nature of fraud attack on each survey, with Survey 2 probably having attracted more sophisticated fraud as evident from responses to open-ended questions.

Nevertheless, there were a small number of indicators with high predictive power in identifying fraud. Our priority was to identify fraud indicators with minimal false positive rates since the deletion of valid responses undermines the effectiveness of survey research. In the following section, we begin with indicators grouped by effectiveness based on their overall indicator quality and conclude with indicator ensembles.

### Effective indicators (very high and high quality indicators)

**Email address score [Test 28]:** observing a discernible pattern in the length and numerical composition of fraudulent versus valid respondents' email addresses led us to develop an email address score (Table 4). This indicator performed exceptionally well in its predictive power and ranked amongst the top in recall. The scoring was based on the revelation that no valid respondents possessed email handles >22 characters in length or containing more than six numerical digits (an email handle is defined as all characters before “@”). In comparison, other studies have used four or more digits (Griffin et al., 2022) and 10 or more digits (Pratt-Chapman et al., 2021) as their threshold. Points were assigned to emails based on the total number of characters, digits and capital letters contained in the email handle. Email addresses with a score ≥2 were flagged in the “suspicious email address” indicator.

**Table 4:** Email address score and structure of fraudulent vs. valid respondents.

	**Fraud**	**Valid**	**Predictive power**	**Points assigned**
	***n* = 1,078**	***n* = 453**		
**Character length before @**				
≥22	4.0%	0.0%	1.00	3
17–21	21.3%	3.8%	0.85	1
< 17	74.7%	96.2%	0.44	0
**Number of digits before @**				
≥6	5.6%	0.0%	1.00	3
3–5	34.0%	7.7%	0.81	1
≤ 2	60.5%	92.3%	0.40	0
**Number of capital letters before @ (excluding all caps)**				
≥4	0.5%	0.0%	1.00	3
3–2	19.4%	1.3%	0.94	1
< 2	80.1%	98.7%	0.45	0
**Number of capital letters after 3rd character before @ (excluding all caps)**				
≥1	20.1%	0.9%	0.96	1
			*Fraud is total score ≥2*	

Our analysis also corroborated previously identified fraudulent email patterns. One notable structure that has been observed as an indicator of fraud by researchers was two capitalized names (Storozuk et al., 2020; Shaw and Cascalheira, 2023; Goodrich et al., 2023; Griffin et al., 2022; Teitcher et al., 2015). Notably, this structure (e.g. JaneDoe@xxx or JohnDSmith@xxx) was not used by any valid respondents but was observed in several fraudulent responses. The only exceptions to this rule were a few valid respondents who capitalized both names *separated by a period* (e.g. Jane.Doe@xxx). A likely reason for this strong indicator is that the software used to generate spam email addresses may use capitalization to mimic authentic names or entities.

Additionally, we observed patterns in the domain name (after “@”). All fraudulent respondents used the “@gmail” or “@yahoo” domains. While Gmail accounts constituted the majority, this trend may not persist due to Google's recent announcement of forthcoming updates to enhance security measures and deter spammers (*New Gmail Protections for a Safer, Less Spammy Inbox*, 2023). Previous studies have found fraud respondents using other host domains such as the apple domain “@me.com” (Dewitt et al., 2018). Meanwhile, we found some valid respondents used unique domain names (such as a business domain or had topically relevant terms, such as “vineyards” or “ranch” within the domain name). Other approaches to email address analysis include flagging email handles with nonsensical combinations of letters not typically used in emails (Lawrence et al., 2023) and those beginning with numerical values or switching between a series of letters and numbers (Ballard et al., 2019; Mitchell et al., 2020).

A significant percentage of fraud (40%) was successfully identified by leveraging our email address scoring approach. Having a quick way to identify fraudulent email accounts allowed us to retain responses that were otherwise difficult to discern for further analysis.

**Suspicious Activity—MinFraud score [Test 13]:** A MinFraud Risk Score above 59 exhibited perfect predictive power, remaining unfailed by valid respondents. It demonstrated good recall, catching 38% of all fraud. Providing as much information as possible to MinFraud (IP address *and* email address) improved the recall of this indicator. This indicator stands out prominently, as it did in Zhang et al.'s (2022) Mturk-based survey tests, where IP MinFraud caught 87% of fraud with 5% false positives; in contrast, our tests yielded 0% false positives but much lower recall (38% vs. 87%). This might be attributed to the fraud source and type.

**Consecutive [Tests 8, 9]:** Among these four tests (6, 7, 8, 9), start or end times within 1 min before and after another respondent (Tests 8, 9) exhibited the highest predictive power. These tests also had some of the highest recall across all indicators, with an average of 57% of fraudulent cases failing these tests. Our findings are consistent with numerous other studies that have identified consecutive submissions as an effective fraud flag (Shaw and Cascalheira, 2023; Bybee et al., 2022; Dewitt et al., 2018; Chandler and Paolacci, 2017; Lawrence et al., 2023; Mitchell et al., 2020; Irish and Saba, 2023). The introduction of “**consecutive starts**” was a novel indicator, not yet documented in existing literature (Test 6, 8).

Notably, more than 30% of all valid submissions occurred within 1 min of each other. We noted a pattern where valid, direct-link responses consistently arrived within 24 h of email blasts, often appearing within minutes of each other. This pattern highlights the importance of strategically timing marketing efforts during survey administration to enhance response rates (choosing the right time of day and day of week for the population being surveyed) and emphasizes the need to separate open distributions in time from closed distributions.

**Improbable responses [Tests 22–25]:** within this category, improbable locations (Tests 22, 23) emerged as the most effective. Test 22 demonstrated outstanding predictive power, with nearly perfect accuracy in flagging fraudulent responses. Recall varied between surveys, with Test 22 flagging 42% of fraud in Survey 1, but only 10% in Survey 2. Improbable location as an indicator has been corroborated in various studies, confirming its effectiveness (Shaw and Cascalheira, 2023; Pratt-Chapman et al., 2021; Goodrich et al., 2023; Guest et al., 2021; Mitchell et al., 2020). The uncommon demographics indicator (Test 24) exhibited high predictive power with moderate recall, flagging 25% of fraud. This indicator has found support in other studies (Pratt-Chapman et al., 2021).

**Start time [Test 5]:** the response start time of day was considered a reliable fraud indicator for our population. Thirty percent of all fraudsters started between 12:00 am and 5:00 am local time while few valid responses (2%) started during this time frame. Other groups have found a similar pattern between 12:00 am and 4:00 am (Pratt-Chapman et al., 2021). However, others have found that the effectiveness of this indicator might vary and depend on the level of sophistication of the fraud attack (Goodrich et al., 2023). This aligns with our observations since the more sophisticated attack (Survey 2) saw more fraud responses throughout the day.

**Validity tests: incentives [Tests 30–31]:** we found that validity tests based on incentives were effective. In particular, opting out of the survey incentive exhibited very high predictive power and very good recall of valid responses. Remarkably, 99% of fraud cases requested an incentive, while only 68% of valid respondents did the same. It is noteworthy that this indicator might be specific to our population of producers. Brainard et al. (2022) suggest that not all fraudsters request incentives, as they may need to conduct test runs to learn how to credibly respond to the survey.

Moreover, none of the fraudulent respondents in Survey 2 opted to receive their survey incentive through Zelle or Venmo (Test 31). Only 1% (*n* = 9) selected PayPal, while the majority chose to receive their incentive as an Amazon gift card, to be delivered via email.

### Moderately effective indicators (moderate quality indicators)

**Duplication [Tests 17, 15, 16]:** these tests performed reasonably well, with duplicate phone numbers (Test 17, Survey 1) appearing as having very high predictive power, albeit poor recall. Duplicate contact information (email and phone) had higher predictive power in Survey 1, but appeared with poor recall for both surveys. Meanwhile, duplicate IP performed much better in Survey 2 than in Survey 1.

**Geolocation [Tests 10–11]:** as expected of producers during the growing season, the majority of our study population was situated within the study region. Ninety-nine percent of valid respondents possessed U.S. IP addresses (Test 10), and 89% had California IP addresses (Test 11). However, despite these patterns, the geolocation indicator exhibited poor recall on a national level, with 95% of fraudulent responses also having US IPs, but had excellent state-level recall as 50% of all fraud had IPs outside of California. Notably, state-level geolocation emerged as a more accurate measure of fraud in Survey 1 compared to Survey 2. With few exceptions (Kennedy et al., 2020; Levi et al., 2022), most recent studies corroborate our findings that geolocation is a poor measure of fraud when used in isolation (LePine et al., 2023; Ballard et al., 2019; Campbell et al., 2022; Kantar, 2023; Zhang et al., 2022).

**Logical inconsistencies [Test 21]:** while mutually exclusive and other logical inconsistencies have been found to be an effective indicator in the literature (Shaw and Cascalheira, 2023; Goodrich et al., 2023; Dupuis et al., 2019), our tests found that they captured only 7% of all fraudulent responses with variable predictive power between the two surveys. However, these tests had very low error rates (1%), making them potentially useful at finding a few fraudulent responses.

### Ineffective indicators (poor and ineffective quality indicators)

**Speeding [Tests 1–3]:** recent literature has highlighted the diminishing effectiveness of speeding as a fraud measure, with failure rates ranging from 80% (Irish and Saba, 2023) to 99% (Ballard et al., 2019) among fraudulent respondents. While Tier 1 speeding may exhibit strong predictive power (as seen by Survey 2), its overall recall tends to be very poor, reflecting the evolving tactics of fraudsters to bypass these tests.

Only 0.3% of all valid respondents failed the Tier 1 speed test, as completing a 30-min survey in under 6 min can be challenging without the use of software (RepData, 2021; Jibunu, 2021). The literature often acknowledges speeding as an adequate measure of data quality (Griffin et al., 2022; Lawrence et al., 2023), suggesting that it retains significance as a data quality metric, even if it does not flag a substantial amount of fraud (Guest et al., 2021).

An alternative and rarely implemented speeding metric involves evaluating page or question duration, especially for complex questions like matrix questions, open-ended queries, or others that require more time. Professional fraudsters have become adept at circumventing conventional speed tests, but they may not yet be adapting their techniques to regulate their pace on matrix questions (Zhang et al., 2022) or other queries that sincere respondents might naturally take longer to answer.

**Consistency checks [Tests 26–27]:** domain knowledge checks have been recognized as some of the most effective fraud indicators in recent literature (Goodrich et al., 2023; Zhang et al., 2022; Lawrence et al., 2023). They are also perceived as resilient to fraud evolution. Given the diversity of the population we surveyed, the implementation of these checks posed a notable challenge. Remarkably, 91% of fraudulent respondents successfully passed the domain knowledge matching questions (Test 27). Ninety-two percent of fraudsters accurately matched the simpler county-zip code match test (Test 26). This underscores the importance of careful selection and testing of domain knowledge checks before their deployment. Furthermore, our findings support the suspicion that higher incentives can attract more sophisticated fraudsters. These individuals may invest considerable effort, including recordkeeping (as evidenced by their ability to match previous survey responses) and research on the survey subject (as observed in these domain knowledge tests). Additionally, our results indicate a considerable evolution in fraud, as previous studies have reported consistency checks to be effective (Pratt-Chapman et al., 2021; Kennedy et al., 2021; Griffin et al., 2022; Guest et al., 2021) and extremely effective (Pozzar et al., 2020; Goodrich et al., 2023) at identifying fraud.

**Suspicious Activity [Test 11, 14]:** VPN had higher predictive power in Survey 2 than in Survey 1, and blacklisted IPs on MxToolbox was completely ineffective as a measure of fraud. The percentage of fraud to pass suspicious IP activity tests have ranged from 21% (Levi et al., 2022) to 93% (Campbell et al., 2022).

**Identical duration in seconds [Test 4]:** this test had varying results for each survey. Other studies have conversely found that this test is an effective measure of fraud (Shaw and Cascalheira, 2023).

**Data quality [Tests 18–20]:** attention checks (Tests 19, 20) were passed by 82% of valid and 83% of fraudulent respondents. All respondents passed the early attention check (Test 19) at higher rates than the later check. While attention checks can help identify inattentive sincere or “lazy” respondents (Kantar, 2023), they are a largely ineffective measure of fraud (Storozuk et al., 2020; Shaw and Cascalheira, 2023; Kennedy et al., 2021; Kantar, 2023; Zhang et al., 2022). In early studies, fraud respondents were more likely to fail these tests (Kennedy et al., 2020). More recently, studies are finding 75% (Shaw and Cascalheira, 2023), 84% (Kennedy et al., 2021) and as high as 98% (Kantar, 2023) of fraud respondents can circumvent attention checks. Straight-lining and zigzag (Test 18) also proved to be ineffective, though performed slightly better in Survey 1, which had three times more matrix questions than Survey 2.

**CAPTCHA:** despite activating the Qualtrics CAPTCHA, a notable volume of fraudulent responses persisted. However, explicit testing of CAPTCHA was infeasible due to Qualtrics' CAPTCHA data being inaccessible to researchers implementing the survey, since it terminates surveys for respondents failing the CAPTCHA assessment. The literature largely corroborates these findings (Goodrich et al., 2023; Lawrence et al., 2023), bots may now be routinely programmed to bypass these conventional data quality checks.

### Indicator ensembles

Indicator ensembles represent a concerted effort to harness the effectiveness of several indicators at once, and none of them demonstrated the ability to achieve an acceptable level of data purity in the final cleaned sample. Table 5 shows performance metrics for all six ensembles.

**Table 5:** Evaluation of six indicator ensembles tested across two surveys.

	**Ensemble tests**	**Survey 1**				**Survey 2**			
**Ensemble ID**	**Indicators used**	**Predictive power**	**Recall (fraud)**	**Error (valid)** ^†^	**Residual fraud** ^‡^	**Predictive power**	**Recall (fraud)**	**Error (valid)** ^†^	**Residual fraud** ^‡^
Ensemble 1	5, 13, 28	0.98	89%	2%	12%	0.97	55%	2%	39%
Ensemble 2	5, 7, 9, 13, 28	0.94	94%	5%	7%	0.85	91%	16%	12%
Ensemble 3	5, 6, 7, 8, 9, 13, 22, 28	0.75	99%	34%	1%	0.76	95%	30%	9%
Ensemble 4^a^	{A(6,7) and B(5,9,13,15,28)} – C(30 + 31)	0.96	91%	4%	10%	0.98	68%	2%	31%
Ensemble 5^b^	{D(1,8,9,13,22,28) or E(5,6,7,11,15)} – C(30,31)	0.90	98%	10%	2%	0.93	96%	7%	6%
Ensemble 6^c^	{F(6,7) or G(5,9,13,15,22,23,28,29)} – C(30,31)	0.95	92%	5%	9%	0.97	71%	2%	29%

Ensembles 1–3 removed all responses flagged by any of the indicators listed for that Ensemble. Ensembles 4–6 combined groups of indicators using logical operators.

^†^Error refers to the percentage of total valid responses removed by a given ensemble.

^‡^Residual Fraud represents what percentage of the remaining sample is fraud after running an ensemble to remove suspected fraud.

^a^Ensemble 4 removed responses that had been flagged by at least one indicator in group A and at least one indicator in group B, excluding any responses flagged by either validity indicator in group C. The logical formula is as follows: Ensemble 4 = [A (≥1) and B(≥1)] and NOT C (≥1).

^b^Ensemble 5 removed all responses that had been flagged by at least one indicator in group D or by at least two indicators in group E, excluding any responses flagged by either validity indicator in group C. The logical formula is as follows: Ensemble 5 = [D(≥1) or E(≥2)] and NOT C(≥1).

^c^Ensemble 6 removed all responses that had been flagged by at least one indicator in group F or by at least one indicator in group G, excluding any responses flagged by either validity indicator in group C. The logical formula is as follows: Ensemble 5 = [D(≥1) or E(≥1)] and NOT C(≥1).

Ensembles 1–3 and 6 show a clear trade-off between predictive power and recall when combining indicators additively, as the more indicators were included, the lower the predictive power but the higher the recall. Under more sophisticated fraud conditions (Survey 2), residual fraud at or above 10% persisted for all ensembles except for ensembles 3 and 5. Even 5% random responses—a response pattern attributed to fraud (Pratt-Chapman et al., 2021)—can have a statistically significant effect on research results (Credé, 2010). Ensemble 5 performed the best overall in achieving low residual fraud with minimal error, but comparing its performance across the two survey samples one can see that the cost of achieving low residual fraud appears to be an increased error rate. *In other words, a large percentage of fraud was successfully removed by some ensembles but only at the expense of discarding a percentage of valid responses*. It's notable that an unacceptable degree of 6% residual fraud can persist even after removing 96% of fraudulent responses. The effect is exacerbated for samples with greater proportions of fraud, which is likely the new norm given current trends (see Figure 1).

## Summary and conclusions

Our study's findings reveal an alarming trend: methods considered effective just 1 year ago are becoming obsolete. Our literature review reveals a significant decline in the usability of responses from online surveys, plummeting from an average of 75% to now 10% usable responses in the last 5 years. Domain knowledge checks, responses to matching questions, and open-ended questions—all previously deemed reliable—are now found to be much less effective. As adaptive-learning AI tools improve, the long-term viability of open-ended questions for fraud detection becomes questionable. Even the best formulated open-ended questions may be insufficient to distinguish valid respondents from sophisticated fraudsters and AI.

A crucial observation emerges: no single fraud indicator, when used in isolation, proves adequate. Out of the 31 indicators tested, none could identify more than 60% of fraud without a high error rate of at least 15%−20%. However, a more promising outcome arises when combining indicators into ensembles, with the best ensemble capturing 96% of fraud with a 7% error rate. Nevertheless, such an error rate can significantly impact research findings—correlating directly to loss of valid responses and underscoring the need for additional approaches. Email address score, MinFraud Score, consecutive submissions, incentive opt-out, improbable location, and survey start times between 12:00 am and 5:00 am emerge as top-performing indicators across both surveys, with email address analysis revealing strong patterns and contributing to an innovative scoring technique.

Notably, larger and guaranteed monetary incentives attract more sophisticated fraudsters. We suspect that the greater financial rewards justify greater expenditures of effort in replicating authentic responses, thereby posing a significant challenge to researchers in distinguishing genuine submissions from fraudulent ones. Post-submission email verification uncovers a range of possible tactics employed by fraudsters, including internet searches, note-taking, record-keeping, and the use of AI and scripts, all with the aim of mimicking legitimate participants. The subtlety, emotional tenor, and consistency of certain fraudulent survey responses suggest that human fraudsters, possibly in collaboration with AI and bots, are involved, highlighting concerns about the underreported prevalence of human fraud compared to bot fraud (Moss et al., 2021). The variation in indicator performance between surveys underscores the risk of offering large, guaranteed incentives (as in Survey 2), as we believe this led directly to more sophisticated fraudulent responses that eluded detection by robust indicator ensembles and manual analysis of open-ended responses.

This level of sophistication emphasizes the ever-evolving nature of the challenge and the need for advanced strategies to effectively combat survey fraud, as researchers confront a burgeoning professional survey scamming industry. Although fraudsters may initially be perceived as malicious, it's crucial to consider the underlying issue of global inequality. In many parts of the world, a reward of 20 dollars—a modest survey incentive in the US—would cover essential living expenses for several days or more. This global socioeconomic context adds complexity to the problem and highlights the interplay between financial motives, global inequities, and ethical dilemmas in the tackling of fraudulent responses to online surveys. Indeed, if well-remunerated work to cover basic needs were an economic right across the world, human survey fraud would likely be reduced.

Given this rapidly evolving landscape, we recommend that survey professionals carefully consider their approach. Survey professionals could avoid open distributions altogether or use them in conjunction with other distribution strategies, such as two-step verification or personalized links to verified contacts (closed distributions), as we have done here. Leveraging valid baseline samples to compare valid and fraudulent respondents can provide invaluable insights into fraud behavior. Indeed, given the lack of reliable and effective fraud cleaning tools, obtaining a subsample of verified valid responses through direct distribution or similar methods is indispensable to the task of identifying fraud. As more researchers employ this comparative approach, additional insights can continue to be generated. If an open online survey is necessary and funds allow, hiring a specialized fraud detection company may be an option (RepData, 2021). These companies focus exclusively on survey fraud detection and continually improve their methodologies. However, our initial tests of these companies' fraud detection services have not produced satisfactory results (detailed findings to be published). Additionally, cost, lack of third-party testing, and methodological transparency should be carefully weighed.

### Recommendations for practitioners

In light of our research findings and the current literature, we provide specific recommendations for professionals who use online surveys and also make recommendations for future research on survey fraud.

#### Pre-deployment

1. **Mixed methods research:** employ mixed and multiple methods to the extent possible to triangulate findings with surveys.
2. **Open-ended questions:** if possible, obtain IRB approval to allow for at least one required open-ended question that is designed exclusively to catch fraud. Craft open-ended questions that elicit 1–2 sentences of written input. In addition, add a three-part open-ended question to easily find duplication across responses (for example “*What are your top three concerns”* with three short response boxes). Use generative AI tools, such as ChatGPT, to see how these questions might be answered by fraudsters. If viable for your population, request email addresses, phone numbers, mailing addresses, and/or other verifiable contact information. If you use questions for assessing fraud in this way, don't use it to answer your research questions so that other responses can still be viewed as anonymous.
3. **Select and pilot survey-specific fraud tests:** examine your survey to identify question combinations that might act as indicators for illogical, contradictory, or improbable responses. Identify questions that leverage domain knowledge. Pilot domain knowledge, logical consistency and open-ended questions before deployment to make sure that the survey population will respond as expected.
4. **Collect paradata:** paradata, or administrative data about the survey, has been shown to be an effective way to detect fraudulent activity (RepData, 2021; Zhang et al., 2022) but practitioners should be aware that paradata needs to be programmed into the survey pre-deployment. Where appropriate, paradata could include timestamps on every survey page, referrer link information, Query strings to track the source of the specific response, IP address, machine time, operating system version and language, browser versions, browser timezone, browser language and browser privacy mode. However, practitioners should take caution to only collect paradata in alignment with any anonymity assurances provided to survey participants.
5. **Baseline comparative sample:** employ a baseline comparative sample using personal links sent to verified contacts (closed distribution) to create a valid subsample, enabling a nuanced understanding of fraudulent behavior within the specific context of each survey. Comparing valid and fraud subsamples, albeit time consuming, is potentially more resilient against fraudsters adapting to it since it provides the investigator insight into the unique behavior of valid versus fraudulent respondents within their particular survey.
6. **Distribution types:** opt for a closed distribution to save time with data cleaning and enhance data quality. Open surveys are most vulnerable to fraud, and accurately detecting fraud can be very time consuming and also not always possible. If verified email lists are unavailable, consider establishing partnerships to facilitate list access, prioritizing list development as a primary step.
7. **Distribution timing:** strategically time marketing efforts during survey administration to enhance response rates (choosing the right time of day and day of week for the population being surveyed) and emphasizes the need to separate open distributions in time from closed distributions to make fraud detection more efficient.
8. **Incentives:** carefully consider the incentive structure. Consider using physical or material incentives, or process incentive payments via post mail or location-restricted platforms (e.g. Venmo or Zelle in the US). These are time-consuming but are potent fraud deterrents.

#### Post-deployment

1. **Focus on open-ended question analysis:** invest time in the analysis of open-ended responses, as they offer valuable insights into detecting fraudulent behavior. Don't assume validity purely based on credible sounding open-ended responses, and use a verified valid response sample as a baseline to compare against potential fraud if possible. Consider using AI detection tools, such as GPTZero.me, to find AI generated responses.
2. **Choose indicators:** survey professionals should carefully select unique combinations of indicators tailored to their specific survey and population. While acknowledging the need for customization and that our indicator tests might perform differently within different populations or survey structures, we provide a set of indicators that have shown promise in our study. Initiate with indicators exhibiting 1.0 predictive power, such as Tier 1 speeding, MinFraud score, and incentive types, to definitively filter fraudulent responses. Subsequently, compile a blend of the most impactful indicators suitable for your survey which may include start time, improbable responses, email address analysis, and consecutive submissions. Fine-tune your indicators by evaluating their performance within closed distributions if available.
3. **Indicator adjustments:** be aware that the strength of a particular indicator might change as the study progresses and fraud adapts to the survey content.
4. **Post-submission verification:** initiate contact with potentially fraudulent respondents through email, seeking confirmation of their previously submitted responses. Look for similar response structure, excessive formality, and text copied directly from survey responses to detect fraud.

### Topics for future research

We anticipate the emergence of a research subfield dedicated to the study of fraud within online research. Specifically, we propose seven topics for future research:

1. A detailed third-party analysis of **for-profit tools** used by fraud detection companies that cater to market research is needed, see RepData (2021), for a list.
2. Investigation into optimal **open-ended question formats** for fraud detection, delving into nuances such as length, phrasing, analysis algorithms and AI detection tools.
3. The use of **machine learning** techniques such as sentiment analysis, polarity analysis, contradiction detection, and topic analysis can be employed to detect fraud by scrutinizing the consistency of responses, identifying suspicious patterns or contradictions in participants' feedback, and unveiling potential anomalies in the sentiment or topics discussed.
4. The use of **statistical methods** to analyze complex, non-linear relationships among fraud indicators and indicator ensembles could potentially enhance fraud detection capabilities.
5. Research on the **characteristics of bulk email accounts** is needed. Future research teams could test our email scoring technique with other populations and use other tools (such as machine learning) to improve on our approach.
6. Exploring **unique question types** that are not readily used in survey research such as image-based questions, heatmaps questions, and domain knowledge checks using image recognition. For example, an intriguing approach could involve deploying a Qualtrics heatmap question to ascertain participant locations, followed by distinct queries about their county, city, or zip code in separate survey sections. While potentially challenging to analyze, this method could be more complicated for fraudsters to pass through.
7. Delving into the world of **fraudulent respondents** themselves presents a compelling if uncertain avenue of investigation. Such research on the fraudsters themselves would potentially disclose the diversity of training, tools and approaches they use. Shedding light on fraudsters' *modus operandi* may enable online survey professionals to develop more targeted countermeasures.

In conclusion, as our study unravels the interplay between distribution strategies, fraud detection indicators, and fraudsters' evolving tactics, it becomes clear that the realm of online survey research is in a constant state of flux, with “whack-a-mole” and “cat-and-mouse” games being appropriate metaphors. An ongoing commitment to innovation and collaboration among online survey professionals is needed to ensure the robustness and reliability of the data collected through online surveys. Adopting mixed and multiple methods for data collection can help triangulate survey results within the other methods, which in turn can reduce the reliance on the survey data alone. In addition, paper or postal mail surveys should be given serious consideration as an alternative or complement to online surveys, where applicable. Paper surveys yield valid responses more reliably and would thus instill greater confidence in data quality compared to online surveys. While paper surveys are expensive to produce, the costs of conducting online surveys are liable to increase significantly, given the challenges posed by fraud in the online environment. If pursuing an open-distribution online survey, be prepared to invest significantly more time than expected on fraud measures and data cleaning. Be aware that without a separate closed distribution, it's possible to end up without *any* usable responses. In an era characterized by rapid technological advancements, large global inequalities, and a lack of economic opportunities for so many, it is imperative to stay ahead of fraudulent practices to preserve the integrity of research outputs and maintain the validity and reliability of survey-based research.

## Data Availability

The data analyzed in this study is subject to the following licenses/restrictions. Data is currently under analysis. Upon completion of the project, all data will be securely stored in a designated cloud storage platform. Access to the data is available to interested parties, and individuals can contact us to receive instructions on how to securely access the dataset. We are committed to providing data availability to anyone seeking access, promoting transparency and openness in research. Requests to access these datasets should be directed to npinzon@ucdavis.edu.
