# Supplementary material for: AI-powered fraud and the erosion of online survey integrity: an analysis of 31 fraud detection strategies
Source: Front Res Metr Anal. 2024 Dec 2;9:1432774. doi: 10.3389/frma.2024.1432774 (PMC11646990; doi:10.3389/frma.2024.1432774)
Supplement: Supplementary file 1 [file Data_Sheet_1.docx]

# **Supplementary Materials S1 Appendix**

## **Identifying sophisticated fraud in Survey 2**

In Survey 2, the distinction between fraud and legitimate responses became difficult to discern, as fraudulent responses demonstrated high levels of relevance, subtlety, and consistency compared to those in Survey 1. Fraudulent respondents in Survey 2 were able to connect their answers between questions, attempting to emulate genuine producers affected by wildfires. For instance, when asked about the differences observed in how the fire moved through their property, one respondent stated:

1. *"There was a loss of fertility since the upper layer was carried away after the wildfire outbreak."* They continued by expressing:
2. *"To increase the production of fertilizers to farmers so as to increase productivity,"* when providing details on how they were impacted. Lastly, they suggested:
3. *"Strongly advice farmers to seek livestock and crop insurance in case of wildfire outbreaks,"* at the end of a section on recovery which contained references to insurance.

Alone, these responses may seem valid, but we suspected this respondent as fraudulent due to their open-ended responses being irrelevant to the questions asked (2), containing grammatical errors (3), and exhibiting modes of expression unlike our target population ("strongly advice farmers," "so as to"). Under closer examination of their responses to the rest of the survey we discovered subtle inconsistencies, as they mentioned their operating income was "devastated" due to wildfires, yet they reported total losses as "500 dollars." They used punctuation and spelled out "dollars," and they claimed to be farming five different crop types which they claimed to be located 500 miles apart. Finally, to verify their fraudulent status with full confidence, they failed the post-submission verification email.
